# Impact of Mobility Impairment on Indirect Costs and Health-Related Quality of Life in Multiple Sclerosis

**DOI:** 10.1371/journal.pone.0054756

**Published:** 2013-01-23

**Authors:** Craig I. Coleman, Matthew F. Sidovar, Matthew S. Roberts, Christine Kohn

**Affiliations:** 1 Department of Pharmacy Practice, University of Connecticut School of Pharmacy, Storrs, Connecticut, United States of America; 2 Department of Pharmacy, Hartford Hospital, Hartford, Connecticut, United States of America; 3 Clinical Development and Medical Affairs, Acorda Therapeutics, Inc., Ardsley, New York, United States of America; Hospital Nacional de Parapléjicos - SESCAM, Spain

## Abstract

This study was conducted to estimate the indirect costs and health-related quality of life (HRQoL) (utilities) of multiple sclerosis (MS) patients in the United States (US), and to determine the impact of worsening mobility on these parameters. In collaboration with the North American Research Committee on Multiple Sclerosis (NARCOMS) registry we conducted a cross-sectional study of participants who completed the biannual update and supplemental spring 2010 survey. Demographic, employment status, income, mobility impairment, and health utility data were collected from a sample of registry participants who met the study criteria and agreed to participate in the supplemental Mobility Study. Mean annual indirect costs per participant in 2011US$ and mean utilities for the population and for cohorts reporting different levels of mobility impairment were estimated. Analyses included 3,484 to 3,611 participants, based on survey completeness. Thirty-seven percent of registrants were not working or attending school and 46.7% of these reported retiring early. Indirect costs per participant per year, not including informal caregiver cost, were estimated at $30,601±31,184. The largest relative increase in indirect costs occurred at earlier mobility impairment stages, regardless of the measure used. Participants’ mean utility score (0.73±0.18) was lower than that of a similarly aged sample from the general US population (0.87). As with indirect costs, larger decrements in utility were seen at earlier mobility impairment stages. These results suggest that mobility impairment may contribute to increases in indirect costs and declines in HRQoL in MS patients.

## Introduction

Multiple Sclerosis (MS) is a chronic and progressive neurologic disease, characterized by diverse symptoms and deficits, which primarily strikes adults between the ages of 18 and 45 years [1]. MS follows a long, unpredictable course, and often leads to substantial disability accumulated over time. Moreover, as MS largely occurs in people of working age, it may have an adverse impact on employment status, work productivity, and health-related quality of life (HRQoL) [1], [2], [3].

Previous MS-related cost-of-illness studies have been published in the medical literature [4]; however, few were conducted within a United States (US) population and provided estimates of indirect costs and preference-based health status or utility (a patient-reported measure of perceived health status with values between 1.0 (perfect health) and 0.0 (death)). Kobelt and colleagues [5], [6] reported the costs of lost productivity (indirect costs) and pain and suffering (intangible costs) to be substantial, ranging on average from $17,581–$22,231 and $15,315 per patient per year, respectively, and to increase with worsening disease severity. This study used the Expanded Disability Status Scale (EDSS) to define worsening disease severity, stratifying patients by scores of 0–3.5, 4–6, and 6.5–9.5. While the EDSS is weighted towards mobility impairment in the middle of its scale, it may not be a robust or optimal measure of mobility impairment [7].

The primary aim of the current analysis was to estimate the indirect and intangible costs of MS in a US population using data from the North American Research Committee on Multiple Sclerosis (NARCOMS) registry [8]. Furthermore, the analysis aimed to determine the impact of worsening mobility on indirect costs and utility for MS patients using measures of mobility impairment.

## Methods

This economic study was conducted in collaboration with the NARCOMS Registry. NARCOMS maintains a registry of about 36,000 MS patients, the largest of its kind in the world, capturing self-reported patient data elicited through an extensive semi-annual health survey [8]. More specifically, survey questions ask about demographic factors, disease history, functionality, co-morbid conditions, healthcare providers, employment and income, symptoms, and disabilities including mobility impairment and quality of life. Starting in 2010, NARCOMS, together with Acorda Therapeutics, Inc., began sending a supplemental semi-annual questionnaire to the subset of registry participants that previously reported a Patient Determined Disease Step (PDDS) [7] score of ≤7 (use of a wheelchair or scooter) to gather additional data regarding their work productivity, HRQoL, health status, utility, and mobility impairment. To be included in this economic study, participants had to have completed both the regular spring 2010 NARCOMS update survey and a supplemental survey administered about 2 months later.

The collection and research use of NARCOMS data is approved by the Institutional Review Board (IRB) at the University of Alabama at Birmingham. A separate approval was obtained from the same IRB for the acquisition of the additional data via the supplemental semi-annual questionnaire. The secondary analyses reported here were reviewed and approved by the IRB at Hartford Hospital and conducted with de-identified datasets.

To assess, categorize, and rank participants with respect to their mobility impairment, the PDDS, 12-item Multiple Sclerosis Walking Scale (MSWS-12), and NARCOMS Performance Scale (PS) for mobility were used. The PDDS is a participant-reported measure that is scored ordinally from 0 (no disability) to 8 (bedbound) [9], and is highly correlated to the EDSS (r = 0.64, p<0.0001) [10]. For this study, PDDS responses were subcategorized as 0–2 (EDSS equivalent of 0–3.5; “No”), 3–6 (EDSS 4.0–6.5; “Moderate”), and ≥7 (EDSS = 7.0−9.5; “Severe/Total”) to characterize mobility impairment. The MSWS-12 includes 12 questions that are rated on a Likert scale ranging from 1 (“Not at all”) to 5 (“Extremely”) [11]. These 12 questions assess different aspects of walking: ability and speed of walk; ability to run; ability to climb and descend stairs; balance and smoothness of gait; and support, effort, and concentration required. Each question was used separately to categorize participants. The NARCOMS PS for mobility categorizes patient mobility as 0 (“normal”), 1 (“minimal gait disability”), 2 (“mild gait disability”), 3 (“occasional support”), 4 (“frequent support”), 5 (“severe gait disability”), and 6 (“total gait disability”) [10]. It correlates strongly with the Timed 25-foot Walk test (r = 0.77, p<0.0001) [10].

Costs were calculated as the mean annual cost per patient per year in 2011 US dollars by extrapolating the 6-month recall NARCOMS survey responses to a full year. The human capital approach was used to estimate indirect costs of MS. For participants who reported that they were working (attending school or college was considered the same as working) but for whom MS caused a reduction in the number of hours or full days worked, such productivity losses were valued using the mean hourly compensation rate for civilian workers in March 2011 (wages plus benefits) of $30.07 [12]. The cost of early retirement due to MS, which was defined as leaving the workforce before the age of 65, was calculated as the US national average per capita loss in annual work compensation including benefits ($62,547) [12].

US-specific utility scores between 1.0 and −0.11, on a scale where 1.0 =  perfect health and 0.0 =  death, were derived using the validated, generic, preference-based EuroQoL (EQ)-5D instrument [13]. The EQ-5D consists of five descriptive questions concerning five domains of HRQoL (mobility, self-care, usual activities, pain/discomfort, anxiety/depression) with participants’ pattern of responses used to derive utility scores. To estimate intangible costs, the difference in EQ-5D utility scores between the NARCOMS study sample and published values from a similarly aged sample from the US general population [13] was calculated to estimate the number of quality-adjusted life years (QALYs) lost during one year (a QALY = utility * length of time). The commonly referenced willingness-to-pay values (WTP) of $50,000 and $100,000 per QALY were then used to estimate intangible costs (QALY * WTP value) per participant per year [14]. Similarly, the intangible costs of gradual decrements in mobility were assessed by calculating differences between neighboring ordinal responses on each of the mobility impairment assessment tools.

Continuous variables were summarized as means and standard deviations (SDs); proportions were calculated for categorical variables. Kruskal-Wallis one-way analysis of variance tests were used to compare continuous variables. No adjustments for multiple comparisons were made. Analyses were performed using SPSS (version 17.0, SPSS Inc., Chicago, Ill).

## Results

Of the 4,288 NARCOMS participants who met the study criteria, a total of 3,728 (86.9%) completed both the regular update and supplemental surveys used in the data analysis; however, analyses included 3,464 to 3,611 participants, based on survey completeness. Key characteristics of these participants are depicted in Table 1. Most participants were female (80.1%), over the age of 40 years (80.7%), and carried the diagnosis of MS for more than a decade (84.9%). Approximately one third of participants were not working or attending school. Only a fifth of participants experienced an MS relapse in the prior six months and about 61% were receiving a disease-modifying drug.

**Table 1:** NARCOMS Participant Demographics (n = 3,728).

Demographic	Proportion of sample
Age (years)	
<40 (referent)	19.3%
40–49	38.0%
50–59	32.2%
60+	10.5%
Gender	
Female	80.1%
Male	19.9%
Currently working	
Yes	63.4%
No	36.6%
Attending physical therapy	
Yes	24.1%
No	75.9%
Disease duration (years)	
<10 (referent)	15.1%
10–19	47.5%
20–29	25.9%
30–39	8.5%
≥40	2.9%
Relapse within previous 6-months	
Yes	20.3%
No	79.7%
Annual household income	
<$15,000 (referent)	8.5%
$15,001–$30,000	14.0%
$30,001–$50,000	17.7%
$50,001–$100,000	25.6%
$100,000+	14.2%
Undeclared	20.0%
Receiving a Disease Modifying Drug	
Yes	60.8%
No	39.2%

NARCOMS = North American Research Committee on Multiple Sclerosis.

Respondents reported a substantial reduction in work productivity. Respondents missing work because of MS reported that their work time was reduced by an average of 9.0±8.6 hours per week, and that they missed an average of 8.2±14.7 full days of work in the six months prior to the survey. Thus, the mean total loss in compensation per employed participant per year in the evaluated NARCOMS population was $1,499±7,419. Of the NARCOMS participants <65 years of age, 43.7% indicated that they had retired early. The cost of early retirement alone was estimated to be $29,101±31,202 per participant per year. Thus, the total indirect cost per participant per year was estimated to be $30,601±31,184.

**Figure 1:**
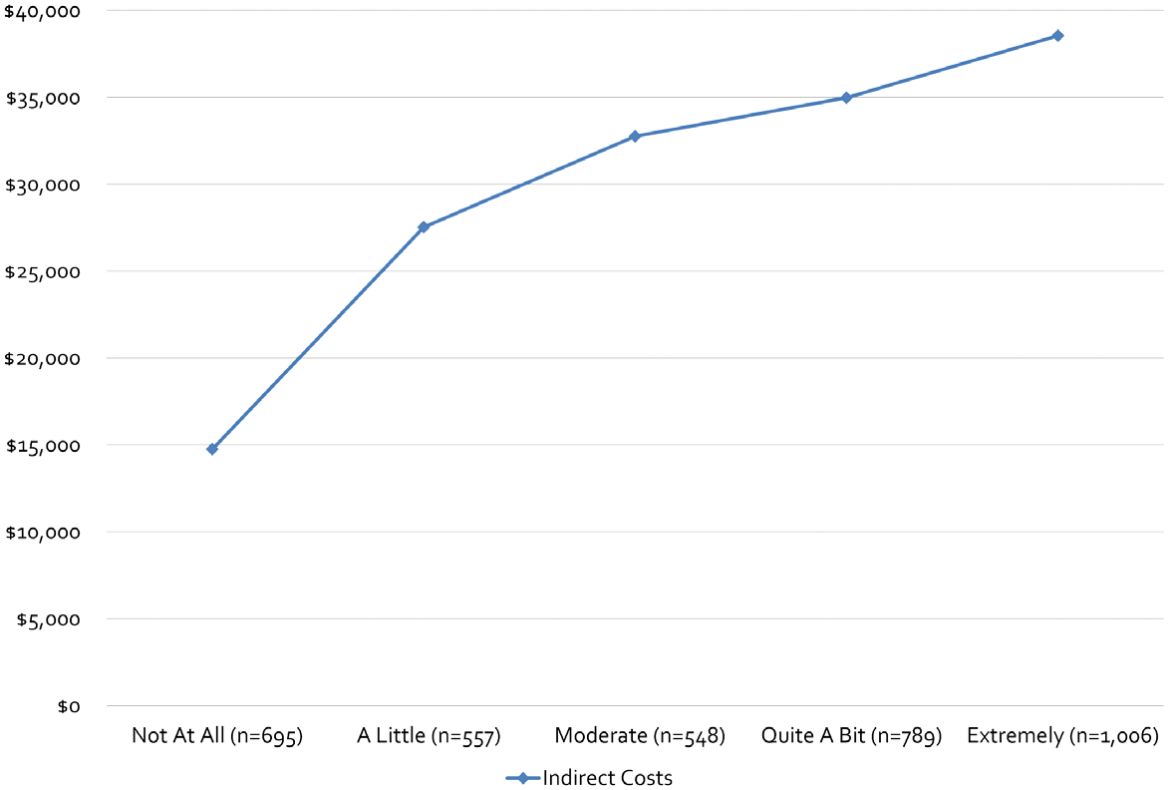
Indirect Costs by 12-Item Multiple Sclerosis Walking Scale Question 6 Responses. MSWS-12 Question 6 =  ”In the past two (2) weeks, how much has your MS limited how far you are able to walk?”.

Tables 2 and 3 present the indirect and intangible costs by different mobility impairment measures. Regardless of the measure used, indirect costs showed a statistically significant trend as mobility impairment worsened, compared to a referent category of no impairment. Moreover, the largest relative increases in indirect costs appeared to occur at earlier mobility impairment stages, typically at the first or second ordinal step in mobility impairment on the various tools considered in this study. Maximum relative changes in indirect costs ranged from 81.6% to 161.3% compared to the referent category of mobility impairment (e.g., “None,” “Normal,” “Not at all”). The smallest total increases in indirect costs were calculated when the PDDS and MSWS-12 questions 8 and 9 (walking indoors and walking outdoors) were used as measures of mobility impairment. The largest were observed when MSWS-12 question 6 (“Limited how far you were able to walk?”) and the Mobility PS were used **(** **Figures 1** **and** **2** **).**

**Figure 2:**
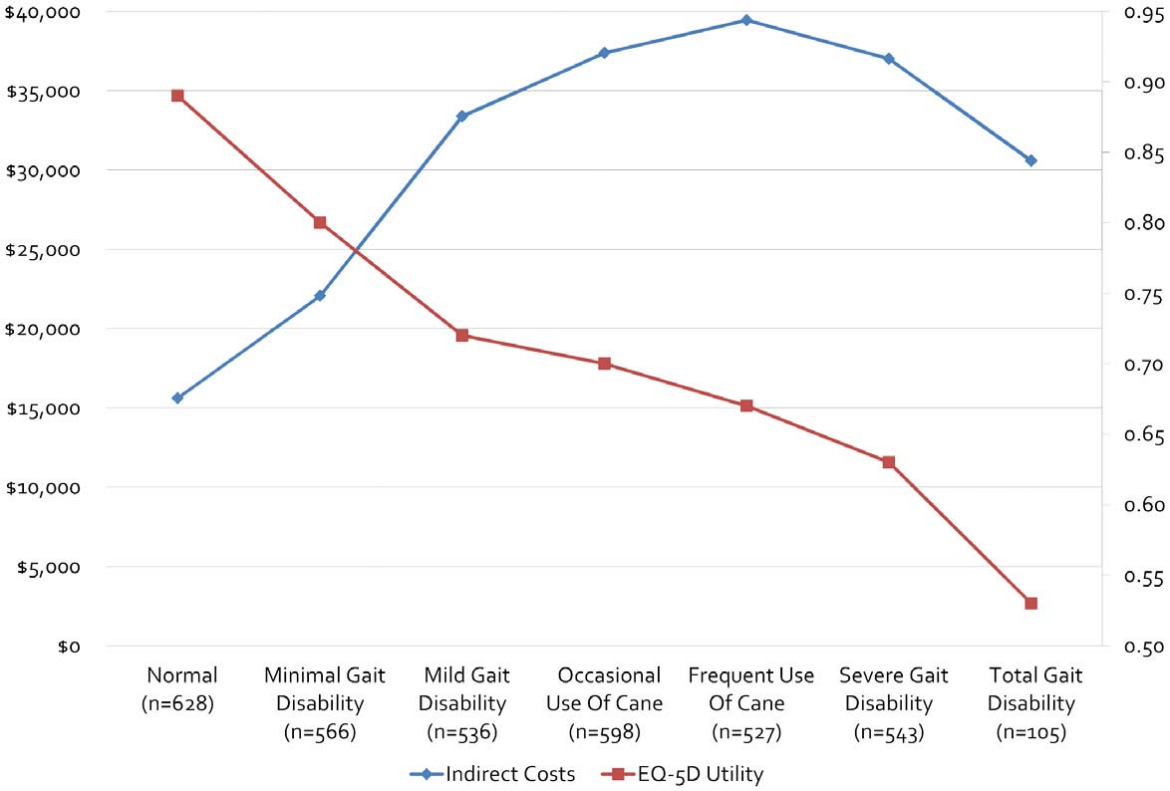
Indirect Costs and EQ-5D Health Utility Scores by NARCOMS Performance Scale Categories. Mobility Performance Scale =  “In the past 4 weeks, compare your current condition to your mobility before you developed MS”.

**Table 2:** Effect of mobility impairment categorized by Patient Determined Disease Steps and the NARCOMS Mobility Performance Scale on indirect and intangible costs#.

**Mobility Impairment**	**Indirect Costs ± SD**	**Change in Indirect Costs (%)** *	**EQ-5D ± SD**	**Change In EQ-5D (%)** *	**Intangible Costs (WTP $50,000, $100,000)**
**PDDS (N = 3,484)**					
None (n = 1,343)	$21,245± $28,808	Referent	0.83±0.14	Referent	Referent
Mild-to-Moderate (n = 1,507)	$ 35,611± $31,533	$14,366 (67.6%)†	0.70±0.16	0.13 (15.7%)†	$6,500, $13,000
Severe-to-Total (n** = **634)	$38,510± $30,291	$17,265 (81.3%)†	0.62±0.19	0.21 (25.3%)†	$10,500, $21,000
**Mobility PS/“In the past 4 weeks, compare your current condition to your mobility before you developed MS” (N = 3,503)**					
Normal (n = 628)	$15,611± $26,556	Referent	0.89±0.12	Referent	Referent
Minimal Gait Disability (n = 566)	$22,055± $29,113	$6,444 (41.3%)†	0.80±0.13	0.09 (11.3%)†	$4,500, $9,000
Mild Gait Disability (n = 536)	$33,380± $31,269	$17,769 (113.8%)†	0.72±0.16	0.17 (23.6%)†	$8,500, $17,000
Occasional use of cane (n = 598)	$37,384± $31,330	$21,733 (139.5%)†	0.70±0.16	0.19 (27.1%)†	$9,500, $19,000
Frequent use of cane (n = 527)	$39,440± $30,199	$23,829 (152.6%)†	0.67±0.17	0.22 (32.8%)†	$11,000, $22,000
Severe Gait Disability (n = 543)	$37,012± $30,824	$21,401 (137.1%)†	0.63±0.18	0.26 (41.3%)†	$13,000, $26,000
Total Gait Disability (n = 105)	$30,576± $31,166	$14,965 (95.8%)†	0.53±0.22	0.36 (67.9%)†	$18,000, $36,000

EQ-5D =  EuroQol 5 Dimension, MS = Multiple Sclerosis, PDDS =  Patient Determined Disease Steps (scores of 0–2 representing “no walking disability”, 3–5 as “mild-to-moderate” and 6–8 as “severe-to-total”), PS =  Performance Scale, Q = question number, SD = standard deviation, WTP =  willingness-to-pay.

* No mobility impairment was used as referent,

† P<0.001.

# Sample sizes for each scale varied due to survey completeness.

**Table 3:** Effect of mobility impairment on indirect and intangible costs#.

Mobility Impairment		Indirect Costs ± SD			Change in Indirect Costs (%)*			EQ-5D ± SD			Change In EQ-5D (%)*			Intangible Costs (WTP $50,000, $100,000)	
**MSWS-12 (Q.1)/”In the past two (2) weeks, how much has your MS limited your ability to walk?” (N = 3,599)**															
Not at All (n = 999)		$18,653± $27,951			Referent			0.87±0.13			Referent			Referent	
A Little (n = 796)		$31,104± $31,337			$12,451 (66.8%)†			0.75±0.14			0.12 (16.0%)†			$6,000, $12,000	
Moderate (n = 668)		$36,412± $30,242			$17,759 (95.2%)†			0.70±0.16			0.17 (24.3%)†			$8,500, $17,000	
Quite a Bit (n = 673)		$37,530± $31,893			$18,877 (101.2%)†			0.66±0.17			0.21 (31.8%)†			$10,500, $21,000	
Extremely (n = 463)		$37,649± $30,140			$18,996 (101.8%)†			0.61±0.19			0.26 (42.6%)†			$13,000, $26,000	
**MSWS-12 (Q.2)/”In the past two (2) weeks, how much has your MS limited your ability to run?” (N = 3,594)**															
Not at All (n = 604)		$16,080± $26,718			Referent			0.89±0.13			Referent			Referent	
A Little (n = 278)		$23,075± $29,441			$6,995 (43.5%)†			0.82±0.12			0.07 (8.5%)†			$3,500, $7,000	
Moderate (n = 222)		$26,082± $29,865			$10,002 (62.2%)†			0.80±0.15			0.09 (11.3%)†			$4,500, $9,000	
Quite a Bit (n = 401)		$32,477± $31,299			$16,397 (102.0%)†			0.76±0.14			0.13 (17.1%)†			$6,500, $13,000	
Extremely (n = 2,089)		$35,999± $31,106			$19,919 (123.9%)†			0.68±0.17			0.21 (30.9%)†			$10,500, $21,000	
**MSWS-12 (Q.3)/”In the past two (2) weeks, how much has your MS limited your ability to climb up and down stairs?” (N = 3,604)**															
Not at All (n = 846)		$17,942± $27,726			Referent			0.88±0.12			Referent			Referent	
A Little (n = 689)		$27,683± $30,907			$9,741 (54.3%)†			0.78±0.15			0.10 (12.8%)†			$5,000, $10,000	
Moderate (n = 678)		$34,423± $30,951			$16,481 (91.9%)†			0.71±0.15			0.17 (23.9%)†			$8,500, $17,000	
Quite a Bit (n = 728)		$36,968± $31,553			$19,026 (106.0%)†			0.68±0.16			0.20 (29.4%)†			$10,000, $20,000	
Extremely (n = 663)		$38,725± $29,950			$20,783 (115.8%)†			0.62±0.19			0.26 (41.9%)†			$13,000, $26,000	
**MSWS-12 (Q.4)/”In the past two (2) weeks, how much has your MS made standing when doing things more difficult?” (N = 3,588)**															
Not at All (n = 967)		$18,204± $18,204			Referent			0.87±0.12			Referent			Referent	
A Little (n = 796)		$29,239± $30,436			$11,035 (60.6%)†			0.77±0.13			0.10 (13.0%)†			$5,000, $10,000	
Moderate (n = 694)		$36,681± $30,373			$18,477 (101.5%)†			0.71±0.15			0.16 (22.5%)†			$8,000, $16,000	
Quite a Bit (n = 661)		$38,047± $30,886			$19,843 (109.0%)†			0.65±0.16			0.22 (33.8%)†			$11,000, $22,000	
Extremely (n = 470)		$38,686± $31,408			$20,482 (112.5%)†			0.59±0.20			0.28 (47.5%)†			$14,000, $18,000	
**Mobility Impairment**			**Indirect Costs ± SD**			**Change in Indirect Costs (%)** *			**EQ-5D ± SD**			**Change In EQ-5D (%)** *			**Intangible Costs (WTP $50,000, $100,000)**
**MSWS-12 (Q.5)/”In the past two (2) weeks, how much has your MS limited your balance when standing or walking?” (N = 3,601)**															
Not at All (n = 704)			$16,450± $26,930			Referent			0.89±0.12			Referent			Referent
A Little (n = 903)			$27,520± $31,017			$11,070 (67.3%)†			0.78±0.13			0.11 (14.1%)†			$5,500, $11,000
Moderate (n = 726)			$35,725± $30,500			$19,275 (117.2%)†			0.72±0.15			0.17 (23.6%)†			$8,500, $17,000
Quite a Bit (n = 722)			$37,618± $30,856			$21,168 (128.7%)†			0.66±0.16			0.23 (34.8%)†			$11,500, $23,000
Extremely (n = 546)			$37,936± $30,971			$21,486 (130.6%)†			0.61±0.19			0.28 (45.9%)†			$14,000, $28,000
**MSWS-12 (Q.6)/”In the past two (2) weeks, how much has your MS limited how far you are able to walk?” (N = 3,595)**															
Not at All (n = 695)			$14,750± $25,924			Referent			0.89±0.12			Referent			Referent
A Little (n = 557)			$27,521± $30,957			$12,771 (86.6%)†			0.79±0.14			0.10 (12.7%)†			$5,000, $10,000
Moderate (n = 548)			$32,747± $30,932			$17,997 (122.0%)†			0.75±0.14			0.14 (18.7%)†			$7,000, $14,000
Quite a Bit (n = 789)			$34,969± $31,449			$20,219 (137.1%)†			0.70±0.16			0.19 (27.1%)†			$9,500, $19,000
Extremely (n = 1,006)			$38,547± $30,340			$23,797 (161.3%)†			0.64±0.18			0.25 (39.1%)†			$12,500, $25,000
**MSWS-12 (Q.7)/”In the past two (2) weeks, how much has your MS increased the effort needed for you to walk?” (N = 3,581)**															
Not at All (n = 757)			$17,924± $27,713			Referent			0.89±0.13			Referent			Referent
A Little (n = 680)			$25,918± $30,593			$7,994 (44.6%)†			0.79±0.13			0.10 (12.7%)†			$5,000, $10,000
Moderate (n = 592)			$35,046± $30,777			$17,122 (95.5%)†			0.73±0.14			0.16 (21.9%)†			$8,000, $16,000
Quite a Bit (n = 770)			$36,971± $31,436			$19,047 (106.3%)†			0.68±0.16			0.21 (30.9%)†			$10,500, $21,000
Extremely (n = 782)			$37,300± $30,501			$19,376 (108.1%)†			0.63±0.18			0.26 (41.3%)†			$13,000, $26,000
**MSWS-12 (Q.8)/”In the past two (2) weeks, how much has your MS made it necessary for you to use support when walking indoors (** ***e.g.*** **, holding on to furniture, using a stick, etc.)?” (N = 3,606)**															
Not at All (n = 1,372)			$21,160± $29,242			Referent			0.84±0.13			Referent			Referent
A Little (n = 647)			$32,767± $30,482			$11,607 (54.9%)†			0.73±0.15			0.11 (15.1%)†			$5,500, $11,000
Moderate (n = 333)			$38,843± $30,603			$17,683 (83.6%)†			0.68±0.15			0.16 (23.5%)†			$8,000, $16,000
Quite a Bit (n = 443)			$38,387± $31,504			$17,227 (81.4%)†			0.67±0.17			0.17 (25.4%)†			$8,500, $17,000
Extremely (n = 811)			$37,154± $30,787			$15,994 (75.6%)†			0.64±0.18			0.20 (31.3%)†			$10,000, $20,000
**Mobility Impairment**	**Indirect Costs ± SD**			**Change in Indirect Costs (%)** *			**EQ-5D ± SD**			**Change In EQ-5D (%)** *			**Intangible Costs (WTP $50,000, $100,000)**		
**MSWS-12 (Q.9)/”In the past two (2) weeks, how much has your MS made it necessary for you to use support when walking outdoors (** ***e.g.*** **, using a stick, a frame, etc.)?” (N = 3,605)**															
Not at All (n = 1,427)	$21,108± $28,947			Referent			0.84±0.14			Referent			Referent		
A Little (n = 401)	$31,249± $30,662			$10,141 (48.0%)†			0.73±0.16			0.11 (15.1%)†			$5,500, $11,000		
Moderate (n = 267)	$40,175± $31,354			$19,067 (90.3%)†			0.69±0.17			0.15 (21.7%)†			$7,500, $15,000		
Quite a Bit (n = 431)	$37,906± $30,819			$16,798 (79.6%)†			0.69±0.16			0.15 (21.7%)†			$7,500, $15,000		
Extremely (n = 1,079)	$37,674± $30,876			$16,566 (78.5%)†			0.65±0.18			0.19 (29.2%)†			$9,500, $19,000		
**MSWS-12 (Q.10)/”In the past two (2) weeks, how much has your MS slowed down your walking?” (N = 3,608)**															
Not at All (n = 648)	$16,119± $26,828			Referent			0.89±0.12			Referent			Referent		
A Little (n = 706)	$25,643± $30,555			$9,524 (59.1%)†			0.80±0.13			0.09 (11.3%)†			$4,500, $9,000		
Moderate (n = 480)	$33,545± $31,110			$17,426 (108.1%)†			0.74±0.14			0.15 (20.3%)†			$7,500, $15,000		
Quite a Bit (n = 701)	$36,837± $31,487			$20,718 (128.5%)†			0.69±0.16			0.20 (29.0%)†			$10,000, $20,000		
Extremely (n = 1,073)	$37,179± $30,374			$21,060 (130.7%)†			0.64±0.18			0.25 (39.1%)†			$12,500, $25,000		
**MSWS-12 (Q.11)/”In the past two (2) weeks, how much has your MS affected how smoothly you walk?” (N = 3,607)**															
Not at All (n = 600)	$17,009± $27,324			Referent			0.90±0.11			Referent			Referent		
A Little (n = 744)	$25,544± $30,496			$6,528 (54.5%)†			0.81±0.13			0.09 (11.1%)†			$4,500, $9,000		
Moderate (n = 478)	$30,978± $30,950			$10,163 (84.9%)†			0.73±0.15			0.17 (23.3%)†			$8,500, $17,000		
Quite a Bit (n = 689)	$37,410± $31,524			$14,101 (117.8%)†			0.69±0.16			0.21 (30.4%)†			$10,500, $21,000		
Extremely (n = 1,096)	$30,628± $30,433			$13,938 (116.5%)†			0.64±0.18			0.26 (40.6%)†			$13,000, $26,000		
**MSWS-12 (Q.12)/”In the past two (2) weeks, how much has your MS made you concentrate on your walking?” (N = 3,611)**															
Not at All (n = 635)	$17,529± $27,538			Referent			0.89±0.12			Referent			Referent		
A Little (n = 627)	$23,824± $29,595			$4,988 (40.0%)^‡^			0.80±0.13			0.09 (11.3%)†			$4,500, $9,000		
Moderate (n = 508)	$33,025± $31,754			$11,094 (89.0%)†			0.73±0.14			0.16 (21.9%)†			$8,000, $16,000		
Quite a Bit (n = 724)	$35,313± $30,941			$11,949 (95.8%)†			0.71±0.16			0.18 (25.4%)†			$9,000, $18,000		
Extremely (n = 1,117)	$37,671± $30,818			$13,845 (111.0%)†			0.64±0.18)			0.25 (39.1%)†			$12,500, $25,000		

EQ-5D =  EuroQol 5 Dimension, MSWS-12 =  Multiple Sclerosis Walking Scale, Q = question number, SD = standard deviation,

WTP =  willingness-to-pay.

* No mobility impairment was used as referent,

† P<0.001.

# Sample sizes for each scale varied due to survey completeness.

The mean utility score for all participants was 0.73±0.18. The MS patients in NARCOMS had lower utility scores compared to a similarly aged sample from the general US population (0.87± a standard error of the mean of 0.01) [14], with an average loss of 0.14 QALYs per participant. Based on WTP thresholds of $50,000 and $100,000 per QALY, intangible costs for the average MS patient in the NARCOMS database were estimated to be $7,000 and $14,000 per year, respectively.

As above, regardless of the measure used, utility decrement showed a statistically significant trend. As the largest decrements in utility scores were seen at early mobility impairment stages, so were the largest increases in intangible costs. Maximum changes in intangible costs were $18,000 and $36,000 compared to a referent category of mobility impairment, depending on the WTP threshold used. The smallest decreases in utility and increases in intangible costs were calculated when the PDDS and MSWS-12 questions 2, 8, and 9 (running, walking indoors, and walking outdoors) were used as measures of mobility impairment. The largest changes were observed when the Mobility PS was used.

## Discussion

We have demonstrated that MS is associated with a large burden to society, mainly due to substantial increases in indirect costs and decreased HRQoL that occur in conjunction with mobility impairment. The total indirect costs of MS were estimated to exceed $30,000 per participant per year and MS patient utility estimates to be dramatically lower than that of a similarly aged sample from the general US population. Moreover, the largest relative increases in indirect costs and utility decrements were seen at earlier mobility impairment stages. The results of our analyses confirm those of other studies that have utilized the EDSS or PDDS to stratify patients by mobility impairment. [2], [3], [4], [5], [6], [15].

The profound effect of mobility impairment on indirect costs and HRQoL in this analysis can, at least in part, be explained by previous research conducted in the NARCOMS registry [16]. Using data from the 2006 and 2007 NARCOMS surveys, Salter and colleagues [16] demonstrated that mobility loss was negatively correlated (r = -0.74, P<0.0001) with patients’ ability to complete instrumental activities of daily living (ADLs), such as the more complex daily tasks including communication and transportation. Since the ability to complete ADLs has been shown to have a major impact on MS patient employment rates and HRQoL, we hypothesize that they may serve as a common thread linking mobility to indirect costs and HRQoL (utility). Of note, similar to the increases in indirect costs and utility decrements seen in our study during early stage mobility impairments, Salter et al. found the association between mobility loss and instrumental ADLs to be greatest in individuals with only mild loss of mobility. Together these results and those of Salter et al. support the hypothesis that patients have more difficulty adjusting to early changes in mobility and consequently have to reduce the level of activity and work in which they engage. Further changes in mobility later in the course of the disease appear to have less impact, suggesting patients have already adjusted to their disease state and developed compensatory strategies to overcome or attenuate limitations.

Kobelt and colleagues [5], [6] have also conducted a cost-of-illness study in the NARCOMS registry. These investigators estimated the overall mean indirect cost of MS per patient per year to be as high as $22,231 (in 2004 US dollars) based upon a 42.3% early retirement rate due to MS, and the overall mean utility score of these patients to be ∼0.70. Moreover, Kobelt reported indirect costs of $10,254, $22,080, $20,194 for an EDSS of 0–3.5, 4–6, 6.5–9.5, respectively, and utility scores of 0.82, 0.68, 0.53 for an EDSS of 0–3.5, 4–6, 6.5–9.5, respectively. In the present study, the percent of MS patients retiring early (46.7%), total and EDSS-stratified indirect costs, and utility estimates are all similar to Kobelt’s after factoring in seven years of work compensation increases (the national average compensation rate rose from $22.63 in Kobelt’s analysis to $30.07 per hour in our own). As both analyses were conducted in the NARCOMS registry using similar methodologies, we believe the similarities in our findings to those of Kobelt’s lend credence to our results.

While not conducted in the NARCOMS database, a host of other MS cost-of-illness analyses, both within and outside of the US, have been published in the medical literature. Unfortunately, like the aforementioned NARCOMS cost-of-illness analysis by Kobelt [5], [6], these other studies used only the EDSS or PDDS to categorize patient disability [2], [15], [17], [18]. While the EDSS and PDDS address mobility impairment in the middle portion of the scale, they are not mobility- or walking-specific measures [7], [17]. Consequently, analyses using these scales to define patient mobility have been met with criticism. Moreover, a systematic review by Patwardhan and colleagues [2] compared the results of published cost-of-illness studies using the EDSS and PDDS to categorize disability, and concluded that there was very little agreement among these studies, in particular regarding costs at higher mobility range EDSS levels. Multiple measures of mobility impairment were used to categorize the extent of mobility loss in our analysis, including the NARCOMS mobility PS and each of the individual questions of the MSWS-12. An important finding of this analysis was that many of these mobility impairment measures suggested more dramatic increases in indirect costs and utility decrements across the spectrum of MS, as compared to the EDSS or PDDS, again highlighting the value of using a more mobility-driven measure. Exceptions to this trend included MSWS-12 questions 8 and 9; however, these questions, like the EDSS or PDDS, focus entirely on the need for support when walking.

The current analysis does have limitations worth noting. First, the NARCOMS registry was utilized as the sole source of data. As NARCOMS participants are asked to self-report data at six-month intervals, responses may be subject to reporting or recall bias [8]. Moreover, despite the size of the registry, these patients may not be representative of MS patients as a whole in the US or in other countries, particularly since bedbound individuals were excluded from the supplemental semi-annual survey. Studies have shown that costs other than direct costs contribute a great deal to the overall costs of having MS [2]. Consequently, the analysis focused on such costs. Unfortunately, another limitation of our analysis was our inability to assess other types of non-direct MS costs, including cost of unpaid or family caregiving and lost leisure time. Despite this, we believe that this analysis of indirect and intangible costs stratified by mobility-specific measures of disability provides important new data to the MS economic literature.

### Conclusions

Mobility impairment may contribute to increases in indirect costs and declines in HRQoL in MS patients.
